# Comparison of Susceptibility Weighted Imaging and TOF-Angiography for the Detection of Thrombi in Acute Stroke

**DOI:** 10.1371/journal.pone.0063459

**Published:** 2013-05-23

**Authors:** Alexander Radbruch, Johanna Mucke, Ferdinand Schweser, Andreas Deistung, Peter Alexander Ringleb, Christian Herbert Ziener, Matthias Roethke, Heinz-Peter Schlemmer, Sabine Heiland, Jürgen R. Reichenbach, Martin Bendszus, Stefan Rohde

**Affiliations:** 1 Department of Neuroradiology, University of Heidelberg, Heidelberg, Germany; 2 Department of Radiology, German Cancer Research Center (DKFZ), Heidelberg, Germany; 3 Medical Physics Group, Institute of Diagnostic and Interventional Radiology I, Jena University Hospital - Friedrich Schiller University Jena, Jena, Germany; 4 Department of Neurology, University of Heidelberg, Heidelberg, Germany; Centre Hospitalier Universitaire Vaudois, Switzerland

## Abstract

**Background and Purpose:**

Time-of-flight (TOF) angiography detects embolic occlusion of arteries in patients with acute ischemic stroke due to the absence of blood flow in the occluded vessel. In contrast, susceptibility weighted imaging (SWI) directly enables intravascular clot visualization due to hypointense susceptibility vessel signs (SVS) in the occluded vessel. The aim of this study was to compare the diagnostic accuracy of both methods to determine vessel occlusion in patients with acute stroke.

**Methods:**

94 patients were included who presented with clinical symptoms for acute stroke and displayed a delay on the time-to-peak perfusion map in the territory of the anterior (ACA), middle (M1, M1/M2, M2/M3) or posterior (PCA) cerebral artery. The frequency of SVS on SWI and vessel occlusion or stenosis on TOF-angiography was compared using the McNemar-Test.

**Results:**

87 of 94 patients displayed a clearly definable SVS on SWI. In 72 patients the SVS was associated with occlusion or stenosis on TOF-angiography. Fifteen patients exclusively displayed SVS on SWI (14 M2/M3, 1 M1), whereas no patient revealed exclusively occlusion or stenosis on TOF-angiography. Sensitivity for detection of embolic occlusion within major vessel segments (M1, M1/M2, ACA, and PCA) did not show any significant difference between both techniques (97% for SWI versus 96% for TOF-angiography) while the sensitivity for detection of embolic occlusion within M2/M3 was significantly different (84% for SWI versus 39% for TOF-angiography, p<0.00012).

**Conclusions:**

SWI and TOF-angiography provide similar sensitivity for central thrombi while SWI is superior for the detection of peripheral thrombi in small arterial vessel segments.

## Introduction

Intracranial artery occlusions of embolic and thrombotic origin are the main causes of acute ischemic stroke. Fast and reliable diagnosis of infarction is crucial for successful recanalization because the outcome of intravenous thrombolysis (IVT) and intra-arterial treatment depends on the time span between onset of symptoms and initiation of therapy [1], [2]. For this purpose thrombus assessment in patients with acute stroke is of major clinical relevance since the location of the thrombus may determine the therapeutic decision.

**Figure 1:**
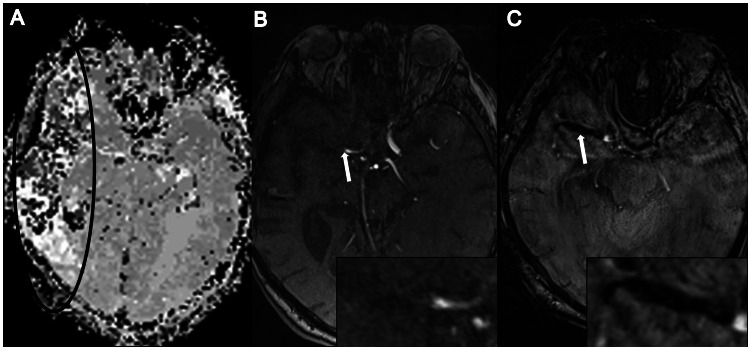
Patient with acute right-hemispheric-stroke. Time-to-peak perfusion map (A) displays impaired perfusion in the area of the right middle cerebral artery (MCA), while TOF-angiography demonstrates occlusion of the right MCA in the M1 segment (B). Typical appearance of a Susceptibility vessel sign (SVS) on SWI is shown in (C).

Time-of-Flight (TOF) angiography is frequently used in routine stroke MR-protocols to image large artery occlusions. In contrast, T2*-weighted sequences are used in acute stroke MRI to image acute or chronic hemorrhages. Moreover, T2*-weighted sequences may detect acute endovascular thromboemboli by identifying a hypointense artifact within the occluded vessel – the so called “susceptibility vessel sign” (SVS) (figure 1) [3], [4]. It is assumed that SVS are mainly caused by the presence of deoxyhemoglobin within the thrombus, even though other components, such as calcifications, may contribute to the appearance of SVS.

Susceptibility weighted imaging (SWI) is a new technique that increases the contrast and conspicuity of susceptibility inclusions, such as deoxyhemoglobin or calcium, by converting the gradient echo (GRE) phase images, which reflect the magnetic field distortion resulting from these susceptibility inclusions, to a special phase mask to combine this mask with the corresponding GRE magnitude image [5]–[7]. Due to the additional phase information SWI is more sensitive to deoxyhemoglobin or calcium than conventional T2*-weighted GRE-magnitude images [8], [9].

In our study we compared the frequency of SVS on SWI with the frequency of embolic occlusion on TOF-angiography in acute stroke patients who were identified by delayed time-to-peak (TTP) perfusion, the current gold-standard for identification of acute ischemic stroke.

## Materials and Methods

### Patients

All investigations were conducted according to the principles expressed in the Declaration of Helsinki. The study was approved by the local ethics committee of the University of Heidelberg (“Ethikkommission Medizinische Fakultät Heidelberg”, ethical statement S-330/2012). Due to the retrospective nature of this study and the poor clinical condition of the majority of the included patients the ethics committee did not require subsequent informed written consent of the included patients. All acute stroke patients who underwent stroke MRI including dynamic susceptibility contrast enhanced perfusion weighted imaging (PWI), TOF angiography and SWI at the University Hospital of Heidelberg Medical Center between September 2009 and March 2012 were eligible for this retrospective analysis. Patients who presented with clinical symptoms for acute stroke and revealed delayed perfusion on the TTP-perfusion map in the area of the anterior (ACA), middle (MCA) or posterior cerebral artery (PCA) were finally included in this study. A total of 94 patients, 44 male (46.8%) matched these inclusion criteria. Clinical and demographic features of all included patients are summarized in Table 1. Stroke onset was defined as the last time point the patient was known to have no neurologic symptoms. Stroke severity was assessed by using the National Institutes of Health Stroke Scale (NIHSS) by a neurologist with NIHSS-certification. All patients had a NIHSS score >2. For all patients NIHSS score and modified Rankin scale were determined at both the time of admission and discharge. In 33 patients images were acquired within 4.6±2.5 hours after onset of symptoms, in 61 cases the time span between stroke onset and hospitalization remained unclear due to the presence of wake-up strokes but had a maximum of 18 hours.

**Table 1:** Patient characteristics.

Variables	Patients
	n = 94
Demographic	
Male, n (%)	44 (46.8)
Female, n (%)	50 (53.2)
Age, years	69.8±14.9
Infarct localization, n (%)	
ACA	1 (1.1)
MCA M1	39 (41.5)
MCA M1/M2	13 (13.8)
MCA M2/M3	31 (33.0)
PCA	10 (10.6)
NIHSS score, mean±σ	
Admission	13.2±6.8
Discharge	10.1±8.3
Modified Rankin scale score (discharge), Median (IQR)	3 (5-2)

### Image Acquisition

Images were acquired during the clinical workup using a 3 Tesla MR system (Magnetom Tim Trio or Verio (identical technical parameters), Siemens Healthcare, Erlangen, Germany) with a 12-channel head-matrix coil. SWI data were recorded with a 3D, fully flow-compensated GRE sequence using the following parameters: echo time (TE) 19.7 ms, repetition time (TR) 27 ms, flip angle 15°, field of view (FoV) 230×230 mm, slice thickness 3 mm, pixel spacing 0.72 and an acquisition time of 2∶16 min. The GRE magnitude and phase images were converted into SW images by the MR-scanner software. Time-of-flight angiography was acquired prior to the application of contrast agent using a 3D sequence with the following parameters: TE 3.69 ms, TR 21 ms flip angle 18°, FoV 200×200 mm, slice thickness 0.64 mm, pixel spacing 0.52 resulting in an acquisition time of 2∶59 min. For dynamic susceptibility contrast perfusion imaging 0,1 mmol/kg gadolinium based contrast medium (gadoterate meglumine, Dotarem®, Guerbet) was administered and images were obtained with a GRE echo planar imaging (EPI) sequence: TE 35 ms, TR 1920 ms, FoV 240×240 mm, slice thickness 5 mm, 75 dynamic scans (0,1 mmol/kg Dotarem®, 3,5 ml/s using a power injector, injection after the third frame), resulting in an acquisition time of 2∶31 min. Maps for different perfusion parameters including cerebral blood volume (CBV), cerebral blood flow (CBF), mean transit time (MTT) and time-to-peak (TTP) were calculated using the software supplied by the manufacturer.

### Image Analysis

Image analysis was performed on our picture archiving and communications system (Centricity PACS, version 3.0.4, GE Healthcare Integrated IT Solutions, Barrington IL) on 2 large-screen high-resolution monitors by two experienced neuroradiologists (A.R and S.R.). The ischemic infarction area was defined as the region of altered signal on the TTP-perfusion map. All patients with reduced perfusion were subsequently assessed for the presence of SVS on SWI within MCA, ACA or PCA and vessel occlusion or stenosis on TOF-angiography in the affected area. Following previous studies, we defined SVS on SWI as the presence of a hypointense signal at the location of a vessel with a diameter exceeding that of the contralateral vessel. Vessel stenosis or occlusion was defined as signal disruption or signal decrease on TOF-angiography (figure 1) [3], [10], [11].

The identification of the ischemic area on the TTP-perfusion map, presence of SVS on SWI, and occlusion or stenosis on TOF-angiography was evaluated via consensus reading by the two readers based on visual inspection.

### Statistical Analysis

Statistical analysis was performed using SPSS 21.0. P values <0.001 were deemed statistically significant. The McNemar-Test was used to compare the frequency of SVS on SWI and occlusion or stenosis on TOF-angiography.

## Results

**Figure 2:**
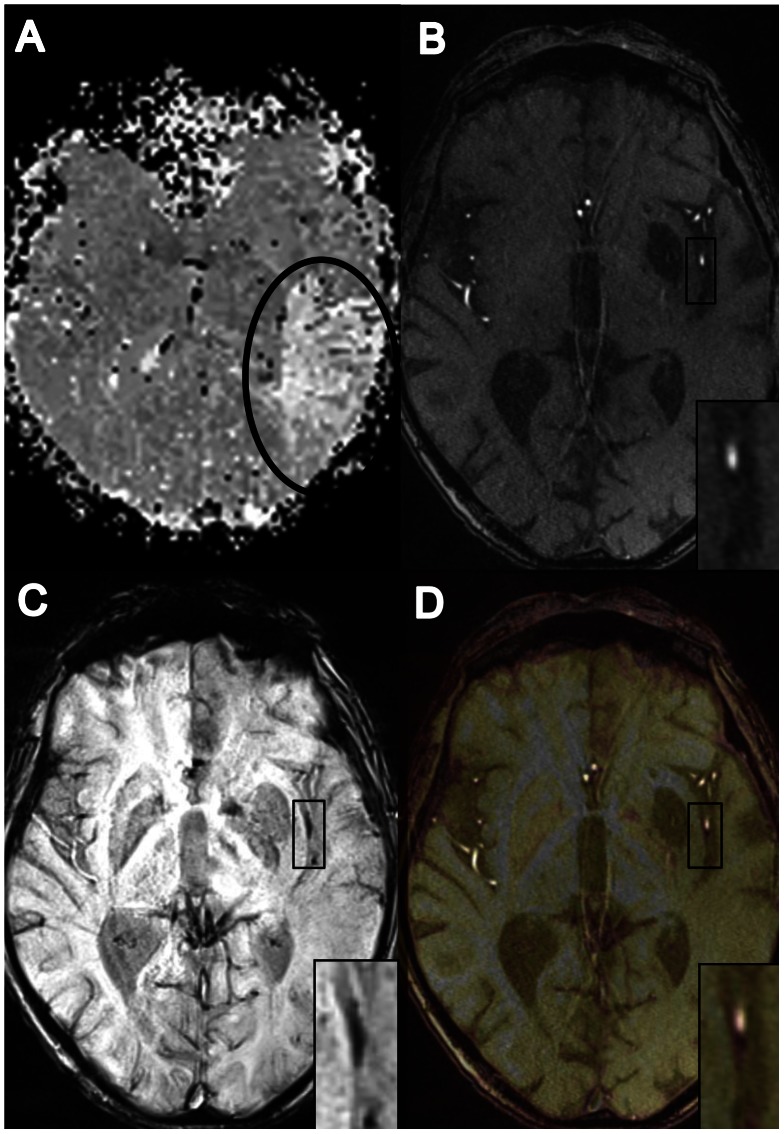
Patient with symptoms of acute stroke presenting SVS without correlation on TOF-Angiography. Diagnosis of acute ischemic stroke based on reduced time-to-peak perfusion (A). On TOF-Angiography (B) no occlusion or stenosis can be detected in left hemispherical arteries. In contrast, the thrombus in the peripheral segment of the MCA is clearly seen on SWI (C). Co-registration of SWI and TOF-angiography data, obtained with the Siemens fusion tool (D), highlights the TOF-angiography signal within the SVS.

Delayed perfusion was observed within the area of ACA (1 patient), PCA (10 patients), M1 (39 patients), M1/M2 (13 patients), and M2/M3 (31 patients). Eighty-seven (92.6%) of the 94 patients presented a correlate on SWI in terms of a clearly delineated SVS within the affected vessel. In 72 patients (76.6%) the SVS displayed an equivalent on TOF-angiography, defined as vessel occlusion or significant stenosis of the corresponding artery (figure 1). Fifteen patients (16.0%) with SVS did not reveal any signal alterations or vessel occlusion/stenosis on TOF-angiography (figure 2). In 7 patients (7.4%) a correlate for acute ischemic stroke could not be observed either on SWI or on TOF-angiography despite clinical symptoms and delayed TTP-perfusion. No cases were found with occlusion or major stenosis on TOF-angiography but absence of SVS on SWI.

**Figure 3:**
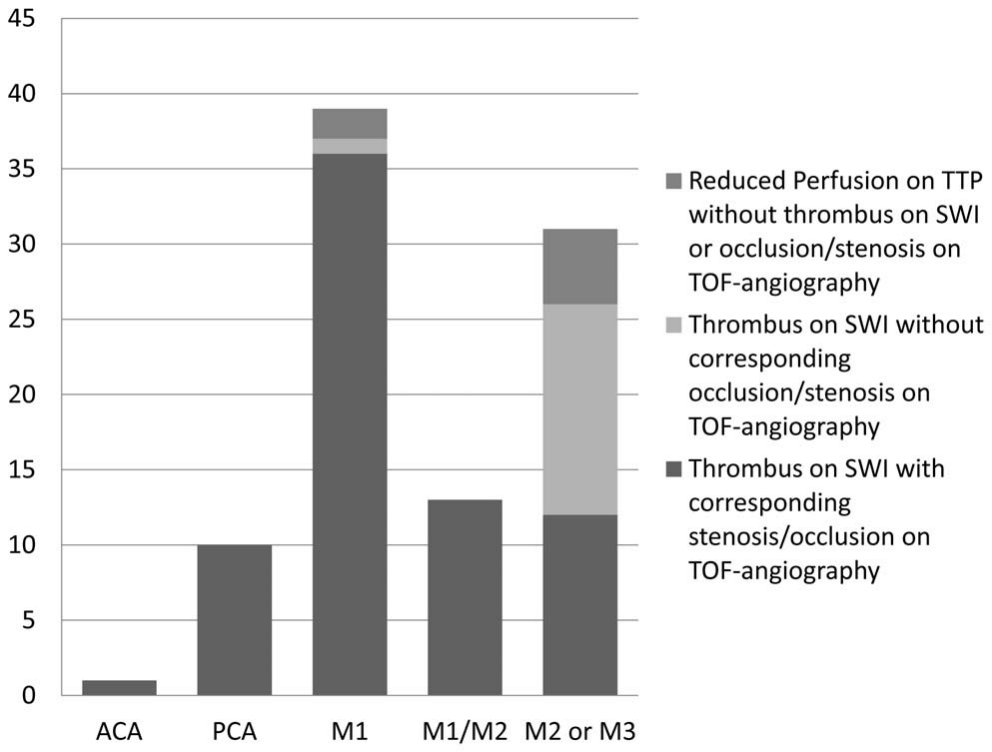
Distribution of observed SVS on SWI and occlusion/stenosis on TOF images in patients with delayed perfusion. 15 of 94 patients presented SVS on SWI without a corresponding occlusion or stenosis on TOF-angiography. In contrast, no patient presented an occlusion or stenosis on TOF-angiography without a corresponding SVS on SWI. Only 7 patients did not present any SVS at all.

Vessel occlusions in patients with both SVS and loss of signal on TOF-angiography (n = 72) were predominantly located in the MCA M1 (50%), MCA M1/M2 (18.0%) and MCA M2 or M3 (16.7%). The PCA was affected in 13.9%, occlusion in the ACA occurred in 1.4% of all cases. In patients with no correlate for vessel occlusion in TOF-angiography (n = 15), the SVS was located in the MCA M2 or M3 (93.3%) and MCA M1 (6.7%). All data are summarized and visualized in figure 3.

Sensitivity for detection of embolic occlusion within M1, M1/M2, ACA and PCA was 96% for TOF-angiography and 97% for SWI, whereas the sensitivity for detecting embolic occlusions within M2 or M3 was 84% for SWI and 39% for TOF-angiography. The McNemar-Test revealed no significant difference between the frequency of SVS on SWI vs. occlusion or stenosis on TOF-angiography for M1, M1/M2, ACA and PCA. In contrast the difference between both techniques for the detection of thrombi within the peripheral segments M2/M3 was significant (p<0.00012).

## Discussion

Our study shows for the first time that SWI is significantly more sensitive than TOF-angiography for detecting peripheral thrombi in patients with acute ischemic stroke.

We found SVS clearly delineated on SWI in 91.6% of all patients with delayed perfusion on TTP. Previous studies that used T2*-weighted GRE sequences for thrombus imaging in patients with acute stroke reported substantially lower rates of SVS between 47.4 and 82% of affected patients [3], [10], [12], [13].

One possible explanation for the increased incidence of SVS on SWI in our study compared to conventional T2*-weighted imaging is the increased field strength of 3 Tesla that is associated with a nearly two-fold SNR than that of 1.5T [14]. The increased spatial resolution, the larger product of the main magnetic field strength and echo time (B_0_-TE product), and the SWI-specific combination of magnitude and phase information employed in this study yielded increased sensitivity towards susceptibility inclusions such as deoxyhemoglobin within the intra-arterial thrombus compared to conventional T2*-weighted imaging [15]. Consequently, we may have detected SVSs that were not visible in the previous studies [12], [13], [3], [10].

The missing SVS in five patients with delayed perfusion on TTP indicative of M2/M3 occlusion and 2 patients with delayed perfusion indicative of M1 occlusion may be due to a small local concentration of deoxyhemoglobin within the thrombus below the limit of detection [13]. Further research is required on the biochemical composition of acute thrombi and its impact on the depiction on SWI [13], [16]. Another reason for the missing SVS could be spontaneous recanalization of the occluded vessel. Previous studies estimated the rate of spontaneous recanalization in acute stroke of the MCA at 12.5% within 290±73 minutes after symptom onset [17]. The remaining 7.4% (seven) of the patients who did not display any SVS are hence within the bounds of the anticipated spontaneous recanalization rate.

The fact that the vast majority of missing SVS were located in the M2/M3 segment indicates that the vessel diameter is a predictor for the appearance of SVS. Since the thrombi occluding M2/M3 have smaller diameters than thrombi occluding M1, they may contain an amount of deoxyhemoglobin below the detection limit.

In contrast to SWI, TOF-angiography visualizes the absence of blood flow and does not allow direct imaging of the occluding clot [18], [19]. One major limitation of non-contrast enhanced TOF-angiography is its insensitivity towards slow flow or slow in-plane flow [20]. These phenomena limit the potential visualization of small arteries, such as M2 and M3. Like SWI, TOF-angiography also benefits from higher field strengths [21]. However, even at 3 Tesla the peripheral M2 and M3 segments often cannot be visualized reliably on TOF-angiography, consequently impeding identification of stenosis or occlusion [21]. Reliable identification of M2 or M3 segments may be additionally hindered by motion artifacts of the patient, since patients with symptoms of acute stroke usually present in a bad clinical condition and are often restless.

**Figure 4:**
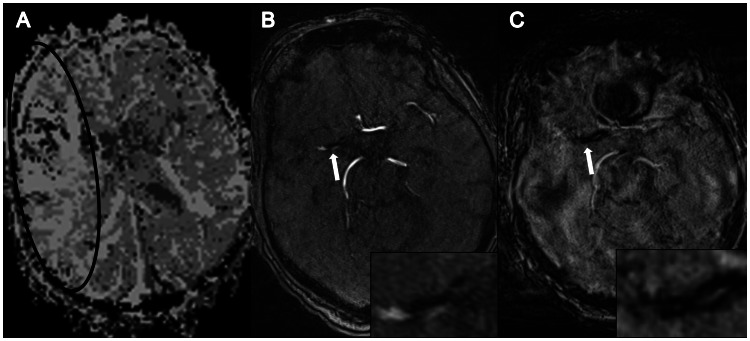
Thrombus delineation despite strong motion artifacts. Patient with symptoms of acute right hemispheric stroke and delayed time-to-peak perfusion in the M1 territory of the right MCA (A). TOF-angiography demonstrates occlusion of the right MCA (B). Despite strong motion artifacts the SVS can still be clearly delineated in the right MCA on SWI (C).

Identification of SVS on SWI is facilitated in vessels with small diameter or in patients producing severe motion artifacts due to the property of SVS to substantially exceed the vessel diameter (figure 2 and 4). This phenomenon is caused by the non-local magnetic field perturbations due to variations in the underlying magnetic susceptibility distribution of the tissue [6], [7]. These field perturbations extend beyond the susceptibility inclusion and manifest themselves as signal dropout in SWI caused by spin dephasing in GRE magnitude images and non-local signal contributions in the GRE phase mask. Hence, the size of the inclusion, e.g., the size of the occluding thrombus, is significantly overestimated on SWI [22], [23]. While this phenomenon is disadvantageous for identifying the real proportions of the thrombus, it enables detection of susceptibility variations in the sub-voxel regime.

It is not expected that SWI will replace TOF-angiography in the management of patients with acute stroke because TOF-angiography provides important additional information for stroke identification and classification due to peripheral vessel rarefication [24]. Rather, both techniques would complement each other for visual identification of the occluded vessel with additional readout parameters. However, acquisition time is a major issue for optimal treatment workflow, since endovascular or pharmacological therapy has to be applied as soon as possible in patients with acute stroke [25]. In our study the SWI sequence parameter setting resulted in an additional acquisition time of only 2∶16 min. Further studies may investigate whether acquisition time in stroke imaging can be reduced by the recently introduced simultaneous acquisition of TOF and SWI [26].

Generally, at least one GRE-sequence is mandatory in acute stroke MRI protocols to detect possible hemorrhagic transformation. Taking into account the reported reliability of thrombus-imaging on SWI, we highly recommend implementation of SWI into routine acute stroke MRI protocols, which comes at no additional cost of measurement time by exploiting the often neglected phase of the complex GRE signal.

A limitation of this study is the variation of time spans between the onset of symptoms and MR imaging of the patients that may result in a loss of accuracy of either one method in terms of thrombus imaging. Furthermore future studies should focus on a comparison of the thrombus on SWI and digital subtraction angiography which directly visualizes the occluding thrombus.

Ultimately, sophisticated post-processing methods exploiting the GRE phase [27]–[30] may contribute further to determine and investigate the exact clot length and identify possible other sources of clot composition, such as calcifications [31].

### Conclusion

SWI provides a reliable technique for thrombus assessment which is superior for the detection of M2 and M3 thrombi compared to TOF-angiography. Hence, we recommend incorporating SWI into the routine imaging protocol for acute stroke.
